# CT imaging biomarkers after neoadjuvant immunotherapy in resectable non-small cell lung cancer: from response assessment to surgical decision-making

**DOI:** 10.3389/fmed.2026.1949375

**Published:** 2026-10-22

**Authors:** Yu Li, Lan Wen

**Affiliations:** The First People’s Hospital of Changde City, Changde, Hunan, China

**Keywords:** computed tomography, dual-energy CT, imaging biomarker, neoadjuvant immunotherapy, pathologic response, radiomics, resectable non-small cell lung cancer, surgical decision-making

## Abstract

Neoadjuvant immune checkpoint blockade, usually combined with platinum-based chemotherapy, has improved pathologic response and event-free survival in patients with resectable non-small cell lung cancer (NSCLC). Preoperative assessment remains difficult, however, because radiographic change does not directly represent the amount of residual viable tumor. Immune-cell infiltration, edema, necrosis, granulomatous nodal reactions, and reparative fibrosis may preserve tumor size despite major or complete pathologic response, whereas small viable tumor nests may remain within an apparently fibrotic bed. This Mini Review examines computed tomography (CT) biomarkers after neoadjuvant immunotherapy, focusing on conventional and dual-energy CT, radiomics, delta-radiomics, and deep-learning models. Size-based response criteria are reproducible but insufficient for tissue characterization. Selected, predominantly retrospective studies suggest that temporal models can add information, but transportability and clinical utility remain uncertain. We distinguish four intended uses: pathologic-response prediction, nodal restaging, technical resectability and operative-complexity assessment, and prognosis. Conventional CT informs anatomy and complication assessment; quantitative response models remain investigational. Suspicious new or enlarging nodes warrant tissue confirmation when the result would alter curative surgery. A structured reporting checklist links imaging uncertainty to multidisciplinary action. Imaging biomarkers should complement clinical, pathologic, and molecular data and should not currently be used to omit surgery.

## 1 Introduction

The treatment of resectable non-small cell lung cancer (NSCLC) is being reshaped by neoadjuvant and perioperative immune checkpoint inhibition. Randomized phase 3 trials support neoadjuvant nivolumab plus chemotherapy (CheckMate 816) and perioperative regimens that also include postoperative immunotherapy (KEYNOTE-671, AEGEAN, CheckMate 77 T, NeoTORCH, and RATIONALE-315) (1–6). NADIM II was a randomized phase 2 trial, not a phase 3 trial (7). Earlier NEOSTAR cohorts separately evaluated immune checkpoint inhibitor (ICI) monotherapy, dual-ICI therapy, and chemotherapy-containing combinations (8, 9). These regimens, and initially resectable versus conversion-treatment populations, are not interchangeable. Stage labels must be interpreted using each study's staging edition; for example, CheckMate 816 enrolled stage IB (tumors ≥4 cm)–IIIA disease using the seventh edition, whereas later perioperative trials commonly used the eighth edition (1–4).

This progress creates a new imaging problem. Before the immunotherapy era, reduction in tumor diameter was commonly treated as a practical surrogate for cytoreduction. After immune checkpoint blockade, the post-treatment lesion may contain variable proportions of viable tumor, necrosis, activated lymphocytes, macrophages, edema, cholesterol clefts, granulation tissue, and fibrosis (10, 11). Consequently, a radiologically stable mass may contain little or no viable tumor, while modest size reduction may coexist with clinically relevant residual disease. New or enlarged mediastinal nodes may also represent a granulomatous nodal immune flare rather than metastatic progression (12).

The presurgical imaging examination must therefore perform several tasks simultaneously: estimate the probability of major pathologic response (MPR) or pathologic complete response (pCR), identify unequivocal progression, restage lymph nodes, determine technical resectability, map the relationship between the tumor and pulmonary vessels or bronchi, detect treatment-related complications, and anticipate operative complexity. Primary surgical data indicate that resection after neoadjuvant chemoimmunotherapy is feasible, but hilar inflammation and fibrosis may increase dissection difficulty (1, 13). This CT-focused review connects imaging uncertainty to concrete surgical actions rather than ranking modalities alone. Its four intended uses—response prediction, nodal restaging, operative assessment, and prognosis—require distinct validation endpoints.

### 1.1 Review approach

A targeted narrative search was updated on 18 September 2026 using PubMed-indexed publications and publisher records. Search combinations included NSCLC, neoadjuvant immunotherapy or chemoimmunotherapy, CT, radiomics, pathologic response, and surgical outcomes. Primary clinical studies published from 2018 onward were prioritized, together with earlier response-assessment standards. Study selection emphasized treatment setting, scan timing, surgical pathology, and independent or prospective testing. Mixed conversion cohorts and postoperative-only inputs were identified separately.

## 2 Mechanistic basis of imaging-pathology discordance

According to the International Association for the Study of Lung Cancer (IASLC), MPR generally denotes ≤10% residual viable tumor in the treated primary-tumor bed; pCR requires no viable tumor in the primary tumor and sampled regional nodes, generally corresponding to ypT0N0 (11). Nodal response should be reported separately. Local versus central review, tumor-bed sampling, and immune-related pathologic response criteria vary between studies (10, 11). These endpoints are determined after surgery and cannot be inferred reliably from size alone. Loss of viable tissue may reduce volume and enhancement, whereas immune infiltration, edema, hemorrhage, and reparative fibrosis may preserve lesion volume or alter attenuation. Small viable nests may remain within a scar (Figure 1). These are interacting treatment-associated processes, not an obligatory sequence initiated by tumor-cell death.

**Figure 1:**
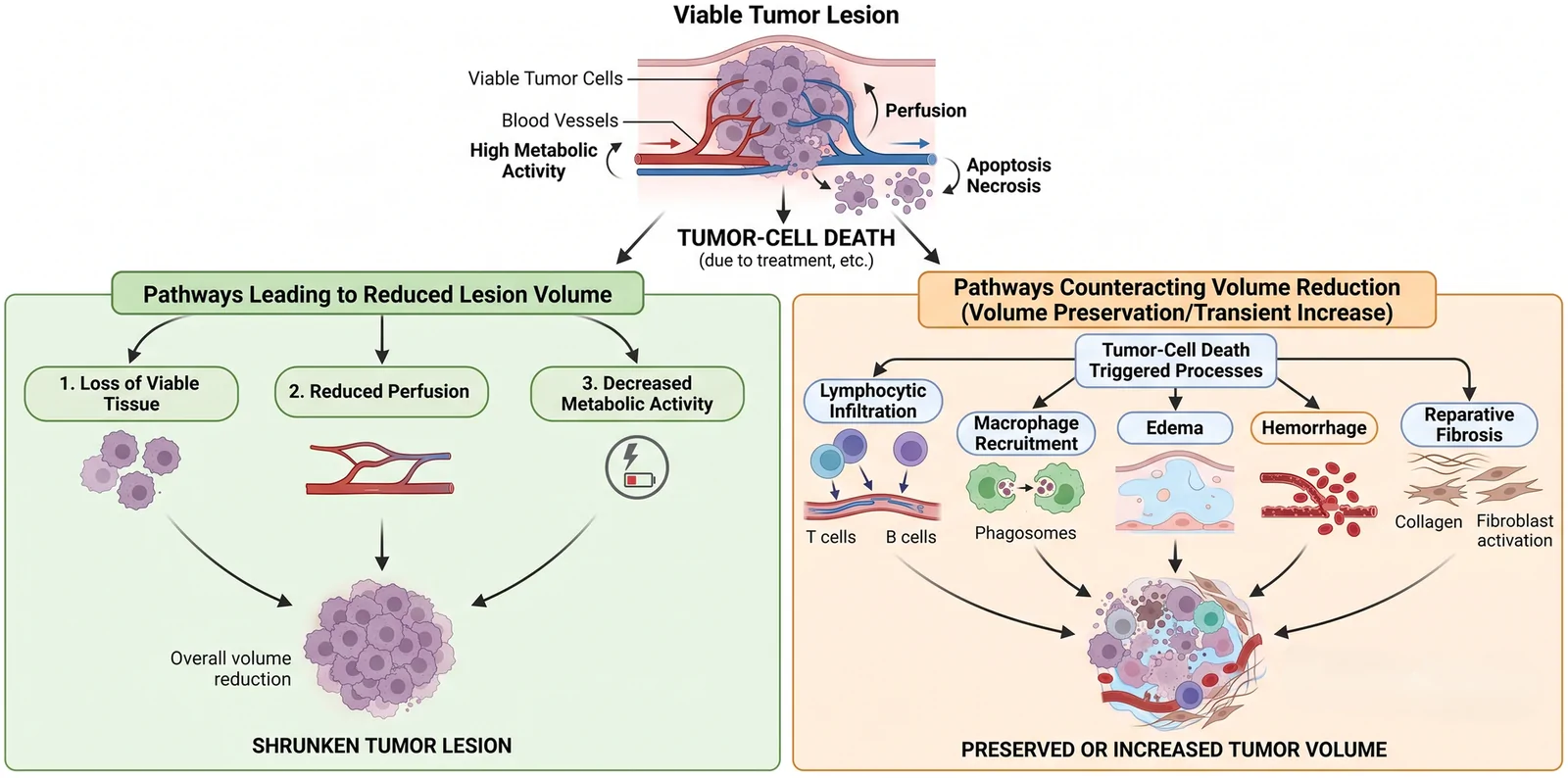
Dual effects of tumor-cell death on lesion volume after neoadjuvant therapy. Loss of viable tumor tissue, perfusion, and metabolic activity promotes lesion shrinkage, whereas immune-cell infiltration, edema, hemorrhage, and reparative fibrosis may preserve or transiently increase lesion volume, contributing to imaging–pathology discordance.

In the NADIM study, post-neoadjuvant circulating tumor DNA (ctDNA) was more closely associated with survival than response assessed by Response Evaluation Criteria in Solid Tumors (RECIST) 1.1, illustrating the biological limits of anatomic response alone (14, 15). Dual-energy CT observations likewise suggest that tumor microenvironmental and perfusion-related features can help explain disagreement between RECIST response and pathologic remission, without establishing an imaging substitute for pathology (16).

Regional lymph nodes add another layer of uncertainty. Immune activation can produce nodal enlargement and granulomatous inflammation without viable metastasis, a phenomenon described as nodal immune flare (12). At the hilum, treatment-associated inflammation and fibrosis may obscure tissue planes around pulmonary arteries, veins, and bronchi. Thus, the same immune-remodeling process that generates a favorable pathologic response can also produce imaging findings that resemble progression and operative findings that increase technical complexity.

## 3 Conventional CT biomarkers: beyond diameter change

Contrast-enhanced chest CT remains central to presurgical intrathoracic restaging and operative-anatomy assessment but does not replace appropriate distant and brain staging. RECIST 1.1 categorization should still be reported as an anatomic descriptor, not a substitute for pathologic response (15). Stable disease according to RECIST 1.1 can include pCR, MPR, and substantial residual tumor. iRECIST allows confirmation of initially unconfirmed progression, usually after 4–8 weeks, but was developed for immunotherapy trials; waiting for this interval may be impractical before planned surgery and should not automatically delay resection (17).

Candidate CT imaging features include percentage change in longest diameter, volumetric reduction, attenuation or density change, development of cavitation, loss of a solid component, restoration of an air bronchogram, and alteration of tumor–vessel or tumor–bronchus interfaces. The presurgical scan should also describe pleural contact, chest-wall invasion, fissural extension, vascular encasement, bronchial narrowing, and the distribution of nodal disease. These anatomical findings inform the feasibility of minimally invasive resection, the potential need for sleeve resection or angioplasty, and preparation for conversion to thoracotomy. However, no single semantic feature has established sufficient accuracy to determine MPR or pCR.

Dual-energy CT offers additional quantitative information through iodine concentration, normalized iodine concentration, effective atomic number, and virtual monoenergetic reconstructions. Iodine-related measurements may reflect perfusion but are also affected by inflammation, contrast timing, and technical factors. A study combining single-source dual-energy CT with tumor-microenvironment features provided exploratory evidence concerning discordance between RECIST 1.1 and pathologic remission (16). Nevertheless, evidence is still limited by single-center designs, variable acquisition protocols, and the absence of universally accepted post-immunotherapy thresholds.

Serial interpretation is more informative than an isolated post-treatment scan. The radiologist should compare pretreatment and presurgical images side by side, document both regression and new inflammatory changes, and explicitly state whether an apparent abnormality is more likely to represent viable tumor, treatment effect, or an indeterminate mixture. This probabilistic interpretation is more useful for multidisciplinary planning than an unqualified response label. Record the intervals from the last treatment to CT and from CT to surgery. Serial measurements require comparable contrast phase, slice thickness, reconstruction kernel, respiratory conditions, and segmentation; primary tumors and nodes should be assessed separately. Data-derived thresholds must be distinguished from prespecified cutoffs. Table 1 summarizes clinical readiness; Table 2 provides representative study-level evidence.

**Table 1:** CT-based approaches, biological signal, and clinical readiness.

Approach and signal	Current contribution	Main limitations/standardization	Readiness for surgical decisions
Conventional contrast-enhanced CT; size and anatomy (12, 15)	RECIST description; nodal targets; vascular/bronchial relationships; complications.	Inflammation and fibrosis can mimic viable tumor. Match contrast phase, slice thickness, reconstruction, and respiratory conditions.	Established for anatomical assessment; not a validated surrogate for MPR/pCR.
Immune-modified response criteria (iRECIST) (17)	Document unconfirmed progression and possible subsequent confirmation.	Usually requires reassessment after 4–8 weeks; trial framework may not fit the presurgical interval.	Contextual aid, not an instruction to delay or omit surgery.
Dual-energy CT; iodine-related measurements (16)	Candidate characterization of perfusion and treatment-associated tissue changes.	Inflammation, contrast phase, iodine normalization, scanner implementation; no accepted universal cutoff.	Exploratory; independent surgical-population validation required.
Radiomics/delta-radiomics; texture, shape, and temporal change (18, 19)	Estimate study-specific probability of MPR or pCR.	Segmentation, feature definition, leakage, endpoint prevalence, and acquisition differences.	Investigational; internal validation is insufficient for transportability.
Deep learning; learned CT representations (20, 21)	Candidate pathologic-response prediction and reader assistance.	Dataset shift, hidden confounding, calibration, and automation bias; prospective cohorts remain limited.	Investigational decision support; no demonstrated basis for surgery omission.

Clinical readiness is a qualitative interpretation of the cited evidence, not a formal grading system. CT, computed tomography; RECIST, response evaluation criteria in solid tumors; MPR, major pathologic response; pCR, pathologic complete response.

**Table 2:** Selected primary studies relevant to CT response assessment and surgical interpretation.

Study/design/cohort	Treatment, imaging timing, and endpoint	Held-out performance or primary clinical result	Validation and interpretive limits
She et al., 2022 (20) Retrospective, 4 centers; *n* = 274.	Neoadjuvant chemoimmunotherapy followed by surgery. Baseline primary-tumor CT within 2 weeks before treatment. Endpoint: MPR.	External AUC: deep-learning score 0.72 (95% CI 0.58–0.85); combined clinical model 0.75 (0.62–0.87). Internal combined AUC 0.77.	Independent external cohort; surgery-selected population. No prospective management-impact test. Additional operating-point metrics were not reproduced from the abstract-level record.
Liu et al., 2023 (18) Retrospective, single center; *n* = 89 (64 training/25 internal validation).	Two to four cycles of chemoimmunotherapy. Baseline noncontrast CT within 30 days before treatment; MPR, confirmed by a senior pathologist.	Validation combined-model AUC 0.81 (95% CI 0.63–0.98); sensitivity 0.75, specificity 0.89, PPV 0.92, NPV 0.67. Calibration plot and Hosmer–Lemeshow *P* = 0.683; DCA presented for training.	Random split, not external validation; small validation cohort and broad stage IB–IIIC inclusion (AJCC 8th edition). Calibration/DCA do not establish clinical impact.
Zheng et al., NeoPred, 2025 (21) Multicenter development/validation with prospective test cohort *n* = 50.	Neoadjuvant chemoimmunotherapy; paired pretreatment and presurgical CT. Endpoint: MPR.	Prospective AUC 0.760 (95% CI 0.628–0.891); reported experts 0.720, AI-assisted experts 0.829.	Prospective reader support signal, but only 50 patients. Specific scan intervals and additional operating-point metrics were not available in the accessible report; no surgery-omission trial.
Zheng et al., 2026 (19) Retrospective, 3 centers; *n* = 263 (106 training/47 internal/110 external).	Stage II–III; predominantly PD-(L)1-based chemoimmunotherapy. Pre/post-treatment noncontrast CT; surgery 4–6 weeks after treatment. Endpoint: pCR.	Combined AUC: development 0.89, internal 0.85, external 0.78 (95% CI 0.69–0.86). External delta-model AUC 0.72; sensitivity 0.64, specificity 0.66. Combined-model DCA: threshold range 0.12–0.55.	Independent external testing; heterogeneous regimens and acquisition. Combined versus delta external AUC difference not significant (*P* = 0.182). Data-derived classification threshold; no prospective utility test.
Cascone et al., 2021 (12) *ad hoc* NEOSTAR analysis; ICI *n* = 44, historical chemotherapy *n* = 28.	Neoadjuvant nivolumab with or without ipilimumab. Baseline/restaging nodal imaging with tissue assessment. Endpoint: nodal immune flare.	Flare in 7/44 (16%) ICI-treated versus 0/28 chemotherapy-treated patients.	Not a diagnostic-accuracy model; historical comparison and small samples. Supports confirmation of management-changing suspicious nodes.
Feldman et al., 2024 (13) Primary NEOSTAR surgical analysis; *n* = 44, with 42 resections.	Nivolumab+chemotherapy (22 patients) or nivolumab+ipilimumab+chemotherapy (22). Endpoint: feasibility and complexity, not CT-predicted response.	Resection 22/22 and 20/22; R0 20/22 and 19/20 operated patients. Challenging resections 22/42; pneumonectomy 3/42. Grade ≥3 complications: 4/22 and 4/20.	Small sequential cohorts. Most operations were thoracotomies (21/22 and 16/20); planned open surgery is not equivalent to conversion. No validated imaging-complexity score.

Metrics retain each publication's endpoint and positive-class convention and are not pooled or directly ranked. PPV and NPV in Liu et al. concern MPR, not absence of residual disease. AUC, area under the receiver operating characteristic curve; CI, confidence interval; PPV/NPV, positive/negative predictive value; DCA, decision curve analysis; ICI, immune checkpoint inhibitor; PD-(L)1, programmed cell death protein 1 or its ligand 1; AJCC, american joint committee on cancer.

## 4 Radiomics, delta-radiomics, and deep learning

Radiomics converts medical images into quantitative descriptors of shape, intensity distribution, texture, and wavelet-transformed heterogeneity. In neoadjuvant chemoimmunotherapy, radiomics is attractive because microscopic viable tumor, immune infiltration, necrosis, and fibrosis may create textural patterns not captured by diameter or visual attenuation. Pretreatment CT models can estimate the probability of subsequent pathologic response, whereas post-treatment models attempt to recognize the residual tumor bed. Delta-radiomics directly quantifies temporal feature changes and is biologically aligned with treatment response.

Retrospective CT models show variable, rather than uniformly high, discrimination (18, 20, 22–25). In She et al. (274 patients), the combined clinical/deep-learning model had an external area under the receiver operating characteristic curve (AUC) of 0.75 (95% confidence interval [CI], 0.62–0.87) for MPR (20). In the 263-patient temporal-radiomics study, combined-model AUC fell from 0.89 in development to 0.78 (95% CI, 0.69–0.86) in external testing; improvement over delta-radiomics was not significant in the external cohort (19). This supports task-specific evaluation, not a general claim of temporal-model superiority. Studies combining neoadjuvant and conversion therapy require separate interpretation (26).

These results should be interpreted cautiously. Surgical-only cohorts create selection and verification bias by excluding progression, toxicity, or unresectability without a resection reference standard. Stage, histology, regimen, tumor size, center, scanner/vendor, reconstruction, and segmentation can confound performance. Internal random splits do not establish transportability; patient-level separation and training-only feature selection, harmonization, and tuning are needed to prevent leakage. Segmentation, image interpretation, and pathology blinding should be documented. External calibration and decision-curve analysis (DCA), together with comparisons against radiologists, RECIST, clinical stage, and simple clinical models, are needed beyond AUC (27–30).

NeoPred illustrates a potentially useful but preliminary direction: in 50 prospectively evaluated patients, dual-time-point CT yielded an AUC of 0.760, compared with a reported expert AUC of 0.720; assisted expert performance reached 0.829 (21). This does not establish deployment readiness. Gan et al.'s framework includes pathomics from resection specimens and therefore cannot be treated as a wholly preoperative tool in that form (31).

## 5 Nodal restaging and imaging-informed surgical decision-making

Surgical decision-making after neoadjuvant immunotherapy should not be reduced to a binary imaging-response assessment. The immediate questions are whether curative resection remains technically possible, whether disease has progressed beyond a surgical field, whether nodal status has changed, and whether treatment has created complications or anatomical changes that alter the planned operation.

Nodal restaging is a high-risk point for misclassification. In an *ad hoc* NEOSTAR analysis, nodal immune flare occurred in 7/44 ICI-treated patients (16%), compared with 0/28 historical chemotherapy-treated patients; these small cohorts do not define a universal incidence (12). If new or enlarging nodes would preclude curative surgery or redirect treatment, endobronchial ultrasound-guided sampling, mediastinoscopy, or another appropriate tissue-confirmation approach should be considered. Radiographic nodal regression does not remove the need for systematic nodal evaluation at surgery.

The presurgical report should characterize the tumor's relationship to the pulmonary artery, veins, lobar and main bronchi, fissures, chest wall, mediastinum, and adjacent lobes. Hilar soft-tissue thickening and loss of fat planes may indicate difficult dissection without proving invasion. In Feldman et al.'s NEOSTAR surgical analysis, 42/44 patients underwent resection, R0 resection was achieved in 20/22 and 19/20 operated patients in the two treatment arms, and 22/42 resections were considered challenging; three pneumonectomies were performed (13).

Imaging findings should be discussed in a multidisciplinary team that includes thoracic surgery, medical oncology, radiology, pulmonology, and pathology, with other specialties as appropriate. Persistent tumor size alone should not justify omission or postponement of surgery when disease remains resectable and there is no verified progression. Likewise, an imaging model that predicts pCR is not sufficiently mature to support nonoperative management. The most defensible current role of CT is to identify targets for invasive restaging, assess complications, anticipate operative anatomy, and support informed surgical planning; quantitative estimates of viable disease remain investigational.

## 6 Integration with molecular biomarkers and future research

CT characterizes imaged disease volume, not the entire microscopic or metastatic burden; spatial resolution, partial-volume effects, and anatomical coverage remain limiting. Tissue biomarkers and ctDNA offer complementary information, as illustrated by NADIM (14). Molecular context must not be confused with validation of an imaging surrogate: a pan-cancer review of DNA damage-response alterations provides biological rationale for treatment heterogeneity (32), whereas the association between NRF2 expression and survival in broader NSCLC cohorts is prognostic evidence, not a neoadjuvant-response or treatment-benefit test (33). A candidate model integrating temporal CT, tissue programmed death-ligand 1 (PD-L1), histology, ctDNA, and clinical stage would still require validation in its intended surgical population.

Related precision-oncology studies further illustrate why molecular context may modify treatment behavior independently of CT phenotype. Work spanning NRF2 prognostic biology in NSCLC, pharmacogenomic targeting of AKT, and CCL19-mediated remodeling of the tumor immune microenvironment demonstrates the breadth of clinically relevant signaling contexts (33–35). Recent mutation- and signaling-focused studies of DNA-damage response alterations, PIK3CA/PI3K*α* targeting, CHD1-associated epigenetic remodeling, MTA1- and Bmi-1-associated stemness and chemoresistance, and RHOJ-driven resistant cell states likewise emphasize molecular heterogeneity across cancers (32, 36–40). These studies provide indirect biological context rather than validation of CT biomarkers, but they support integrating imaging with molecular and immune information in future multimodal models.

Prospective studies should predefine the clinical decision that the biomarker is intended to improve. MPR/pCR prediction, mediastinal restaging, operative difficulty, and recurrence prognosis require different endpoints; predicting benefit from a particular treatment additionally requires appropriate comparative evidence. Acquisition and timing should be harmonized, lesions and nodes segmented reproducibly, and independent validation should include scanners, institutions, histologic subtypes, and regimens not represented in development. Calibration, DCA, subgroup performance, and failure cases are as important as discrimination (27–29).

The most useful future CT biomarker may not be a single number. A structured signature combining temporal morphology, iodine-related measurements, peritumoral remodeling, and nodal behavior could provide complementary information. Priorities are prospective inclusion of patients who do not reach surgery, reproducibility across acquisition protocols, and testing whether model-assisted interpretation changes management safely. A narrative review cannot resolve selective reporting or the heterogeneity of existing cohorts.

For translation, radiomics studies should follow reporting and reproducibility standards. The CheckList for EvaluAtion of Radiomics research (CLEAR) provides radiomics-specific reporting requirements, the Checklist for Artificial Intelligence in Medical Imaging (CLAIM) addresses artificial-intelligence studies in medical imaging, and the TRIPOD+AI statement covers prediction-model reporting (27–29). Deep-learning studies should additionally document preprocessing, architecture, code or model availability, and reproducibility safeguards (34, 41).

## 7 Conclusion

CT after neoadjuvant immunotherapy in resectable NSCLC must assess residual-disease uncertainty while defining nodal status, complications, and surgical anatomy. Inflammation, necrosis, edema, granulomatous reactions, and fibrosis can obscure the relationship between lesion size and viable tumor. Conventional anatomical assessment is clinically established, whereas dual-energy measurements, radiomics, and deep-learning response models remain investigational. External discrimination alone does not establish clinical utility or justify surgery omission. The immediate priority is a structured report linked to tissue confirmation when management would change and to multidisciplinary surgical planning, supported by prospective validation and calibrated decision-support models.
